# METTL3 inhibition alleviates neuroinflammation and apoptosis by reducing ETV4 m6A modification

**DOI:** 10.3389/fimmu.2025.1717319

**Published:** 2026-01-12

**Authors:** Dong He, Xiaokun Jiang, Gengyin Guo, Jinfeng Ma, Jinyan Chen, Yongfei Zhang, Jianfeng Zhuang, Ping Xie, Zhen Zhang

**Affiliations:** 1 Department of Neurosurgery, Shandong Provincial Hospital affiliated with Shandong First Medical University, Jinan, Shandong, China; 2 Shandong Provincial Key Laboratory of Carbohydrate and Glycoconjugate Drugs, Shandong Freda Pharmaceutical Group Co., Ltd., Jinan, Shandong, China; 3 Department of Cell Biology, Laboratory for Clinical Medicine, Capital Medical University, Beijing, China; 4 Department of Neurosurgery, Central Hospital Affiliated with Shandong First Medical University, Jinan, Shandong, China; 5 Department of Neurosurgery, Qilu Hospital, Cheeloo College of Medicine and Institute of Brain and Brain-Inspired Science, Shandong University, Jinan, China

**Keywords:** apoptosis, Etv4, m6A modification, METTL3, neuroinflammation

## Abstract

Intracerebral hemorrhage (ICH) triggers devastating secondary brain injury driven by maladaptive microglial activation and neuroinflammation. While N6-methyladenosine (m6A) RNA methylation influences inflammation, its spatiotemporal regulation in ICH microglia remains unclear. Here, we identified METTL3 as a key epigenetic driver that promotes neuropathology post-ICH. Our analyses revealed that upregulated METTL3 expression in activated microglia in ICH model mice was correlated with increased global m6A levels. Functional studies have demonstrated that METTL3 depletion attenuates the release of proinflammatory cytokines (TNF-α, IL-1β, and IL-6), suppresses NF-κB activation, and reduces apoptosis in microglia. Mechanistically, MeRIP-seq and RNA-seq identified the transcription factor ETV4 as a METTL3 target, where METTL3-mediated m6A modification of the ETV4 3′-UTR recruits the reader IGF2BP2 to increase mRNA stability. This axis drives NF-κB-mediated inflammation and caspase-3-dependent apoptosis. Overall, our work reveals the role of METTL3 in sustaining neuroinflammation and inducing apoptosis via m6A/ETV4 stabilization and suggests that METTL3 inhibition is a promising strategy for ameliorating ICH injury.

## Introduction

Epigenetic modifications play pivotal roles in the regulation of gene expression and are closely linked to disease pathogenesis. Among these modifications, N6-methyladenosine (m6A) is the most abundant, dynamic, and reversible internal modification found in messenger RNA (mRNA) and other RNA molecules in eukaryotic organisms (1–3). This complex regulatory system relies on the coordinated interaction of three distinct groups of proteins: writers (methyltransferases, primarily the METTL3-METTL14-WTAP complex), erasers (demethylases such as FTO and ALKBH5), and readers (a diverse set including YTHDF1/2/3, YTHDC1/2, and IGF2BPs that recognize the m6A mark) (4, 5). The site-specific placement of m6A has a profound effect on various aspects of RNA metabolism, such as mRNA splicing, nuclear export, stability, translational efficiency, and localization (6). As a result, m6A modifications act as essential epigenetic switches that regulate a wide range of biological processes, spanning development, stem cell differentiation, immune responses, and disease progression.

Intracerebral hemorrhage (ICH) is a particularly devastating form of cerebrovascular injury characterized by the abrupt extravasation of blood into the brain parenchyma (7, 8); its pathophysiology is marked by a complex cascade of events: initial hematoma expansion exerts mass effects and direct tissue damage, followed by the cytotoxic effects of blood breakdown products (such as hemoglobin, heme, and iron), overwhelming oxidative stress, robust neuroinflammatory cascades, and blood–brain barrier (BBB) disruption (9, 10). These processes involve intricate and often deleterious interactions between destructive mechanisms and endogenous repair efforts. Central to this neuroinflammatory milieu are microglia, the resident innate immune sentinels of the central nervous system (CNS). Microglia exhibit remarkable yet dual-edged functional plasticity in response to ICH (11, 12). During the acute phase, they contribute beneficially through the phagocytic clearance of hematoma components and cellular debris. However, as injury evolves, microglia can undergo maladaptive activation, often associated with a transition toward a predominantly proinflammatory, ‘M1-like’ phenotype (13, 14). This shift leads to the excessive and sustained release of cytotoxic mediators, including interleukin-1β (IL-1β), tumor necrosis factor-α (TNF-α), reactive oxygen species (ROS), and matrix metalloproteinases (MMPs), which exacerbate secondary brain injury by promoting neuronal death, amplifying neuroinflammation, and further compromising the BBB (15–19).

Emerging evidence underscores the significant involvement of m6A RNA methylation dynamics in the intricate pathophysiology of ICH (20). The dysregulation of m6A modifiers appears to be a crucial epigenetic driver influencing key cellular responses. For example, research has demonstrated that the expression of METTL3, the core catalytic component of the m6A writer complex, is upregulated in microglia following ICH (21, 22). METTL3-mediated m6A modification specifically enhances the stability and translation efficiency of basic leucine zipper ATF-like transcription factor (BATF) mRNA. BATF, a potent transcription factor, subsequently drives the expression of proinflammatory cytokine genes, thereby amplifying the neuroinflammatory response during the acute phase of ICH injury (23). Conversely, the m6A eraser ALKBH5 plays a counterregulatory role. ALKBH5-dependent demethylation has been shown to suppress proinflammatory signaling pathways in microglia, potentially by reducing the stability of transcripts encoding inflammatory mediators or by modulating the activity of key signaling molecules (24, 25). However, the precise spatiotemporal regulatory mechanisms governing ALKBH5 expression and activity across different phases of ICH (acute, subacute, and recovery) and within specific brain regions or microglial subpopulations remain incompletely understood and represent a critical area of ongoing investigation. Notably, unresolved questions persist regarding the temporal–spatial specificity of m6A modifications across different phases of ICH and the precise molecular switches governing microglial polarization balance. Addressing these knowledge gaps holds transformative potential for developing therapies that harness m6A-mediated epigenetic regulation to mitigate secondary brain injury while preserving the neuroprotective functions of microglia.

Therefore, identifying the key m6A-modified “hub” RNAs and their interacting readers that dictate microglial fate decisions is essential. Here, we identified a novel mechanism in ICH in which microglial METTL3 enhances ETV4 stability through IGF2BP2-dependent m6A modification, thereby driving abnormal microglial activation, inflammatory imbalance, impaired M2 polarization, and increased apoptosis, ultimately exacerbating the pathological progression of cerebral hemorrhage.

## Results

### Pathological stimuli from intracerebral hemorrhage trigger the upregulation of METTL3 expression in microglia

**Figure 1:**
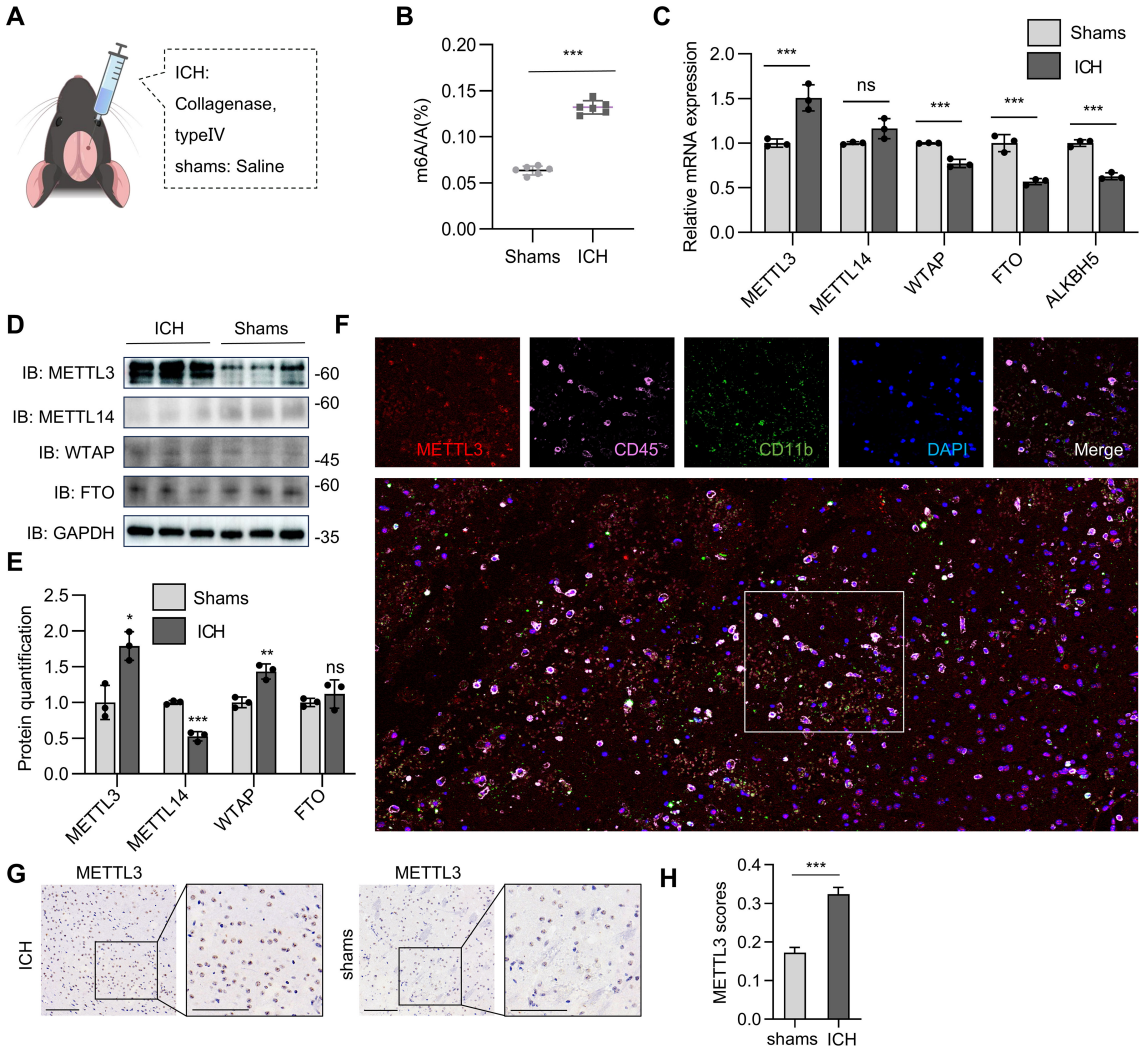
METTL3 upregulation largely mediated the global m6A RNA methylation rise in collagenase-induced ICH. **(A)** A schematic illustration of the Intracerebral hemorrhage **(ICH)** mice model. **(B)** An m6A ELISA assessed m6A mRNA methylation in mouse models of collagenase-induced ICH (n=3 biological replicates of mice, t test). **(C)** Real-time PCR analysis of METTL3, METTL14, WTAP, FTO, and ALKBH5 in collagenase-induced ICH models (n = 3 biological replicates of mice, t test). **(D, E)** Western blot analysis of the protein level of METTL3, METTL14, WTAP, and FTO. **(D)**, the the band density of METTL3, METTL14, WTAP, and FTO was quantified by Image J (n=3 biological replicates of mice, *t-t*est) **(E)**. **(F)** Representative image from immunofluorescence staining of METTL3, CD45, CD11b and DAPI in the brain tissue of mouse model of collagenase-induced ICH. Scale bars, 40 μm. **(G, H)** Representative immunohistochemical (IHC) staining data of METTL3 in brain tissues of ICH mice and sham-operated mice. Scale bars, 90 μm **(G)**, and the expression scores are shown as bar plots. Image J was used to perform semiquantitative analysis **(H)**. Statistical significance is indicated as follows: *: p < 0.05, **: p < 0.01, ***: p < 0.001.

To investigate the relationship between intracerebral hemorrhage (ICH) and m6A methylation, six-week-old male C57BL/6J mice were randomized into two groups (n=3/group). The ICH model was established by the intracerebral injection of collagenase type IV (0.075 U in 0.5 μL of saline) into the right striatum (coordinates: 0.8 mm anterior, 2.0 mm lateral, and 3.5 mm ventral to the bregma). The control mice received an equal volume of sterile saline. At 72 hours postinjection, the mice were euthanized by an anesthetic overdose (pentobarbital 100 mg/kg) for brain tissue collection (**Figure 1A**). Using an m6A enzyme-linked immunosorbent assay (ELISA), we found that m6A RNA modifications were substantially elevated in collagenase-induced intracerebral hemorrhage (**Figure 1B**). The mRNA expression of methyltransferases (METTL3, METTL14, and WTAP) and demethyltransferases (FTO and ALKBH5) was subsequently analyzed (**Figure 1C**). METTL3 mRNA expression was higher in mice with collagenase-induced intracerebral hemorrhage than in controls, whereas WTAP, FTO and ALKBH5 expression was lower in mice with collagenase-induced intracerebral hemorrhage than in controls (**Figure 1C**). Furthermore, the protein abundance of METTL3 was also higher and that of METTL14 was lower in mice with collagenase-induced intracerebral hemorrhage than in controls (**Figures 1D, E**). We did not detect consistent changes in WTAP mRNA expression or protein abundance in this model (**Figure 1C–E**). In addition, multiplex immunofluorescence analysis revealed the marked upregulation of METTL3 expression in microglia-derived macrophages (**Figure 1F**). METTL3 was also found to be increased in the brains of ICH model mice (**Figures 1G, H**). Overall, collagenase-induced ICH increased global m6A RNA methylation, an effect that was driven primarily by upregulated METTL3 expression.

### c-Jun-driven METTL3 upregulation in collagenase-induced ICH

**Figure 2:**
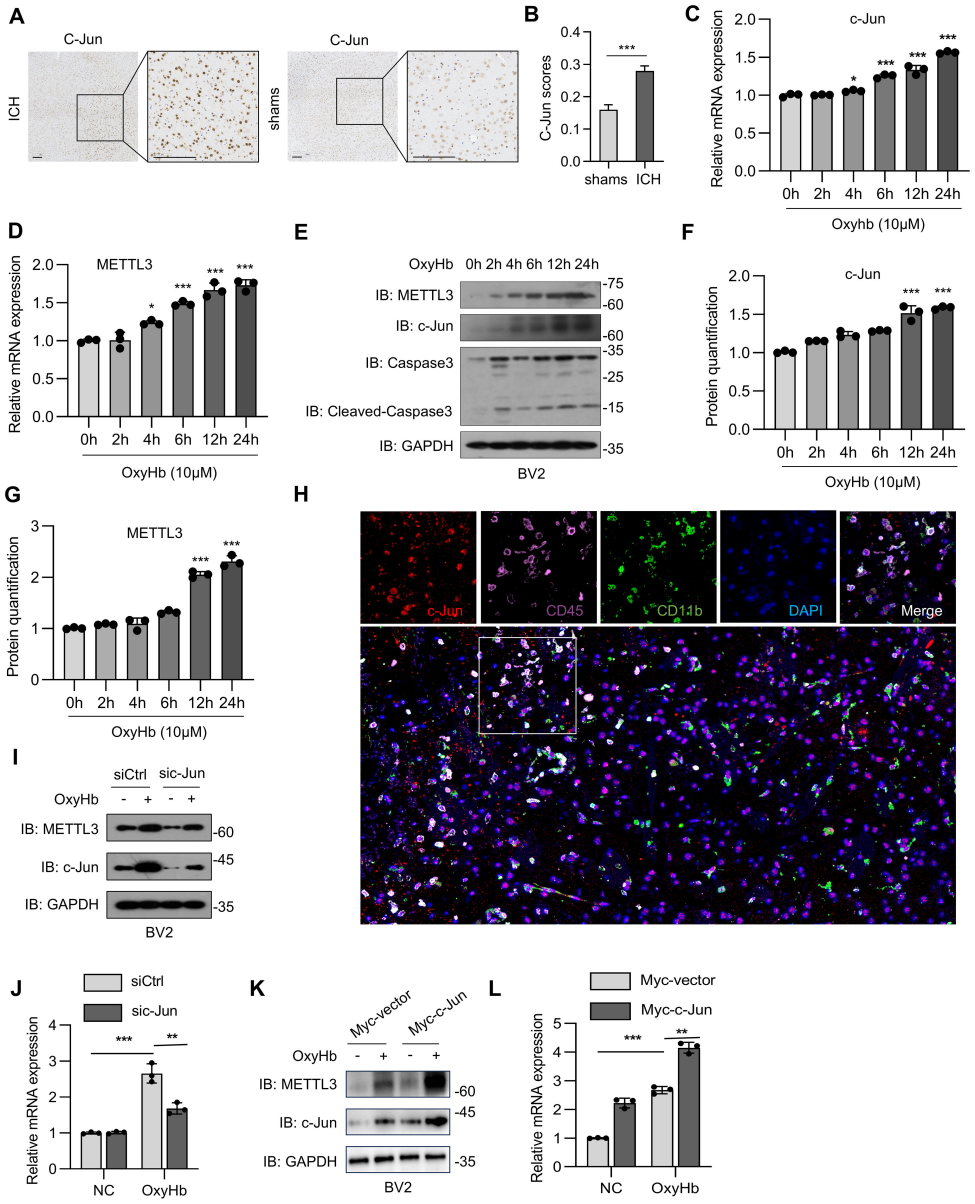
c-Jun drives METTL3 upregulation in microglia. **(A, B)** Representative immunohistochemical (IHC) staining data of c-Jun in brain tissues of ICH mice and sham-operated mice. Scale bars, 90 μm **(A)**, and the expression scores are shown as bar plots. Image J was used to perform semiquantitative analysis **(B)**. **(C, D)** Real-time PCR analysis of c-Jun **(C)** and METTL3 **(D)** mRNA expression over time in oxyHb-treated BV2 cells (n=3 biological replicates of mice, one-way ANOVA). **(E–G)** Western blot analysis of METTL3, c-Jun, Caspase3, and Cleaved-Caspase3 **(E)**, the the band density of c-Jun **(F)** and METTL3 **(G)** was quantified by Image J (n=3 biological replicates, one-way ANOVA). **(H)** Representative image from immunofluorescence staining of c-Jun, CD45, CD11b and DAPI in the brain tissue of mouse model of collagenase-induced ICH. Scale bars, 40 μm. **(I)** Protein abundance of c-Jun and METTL3 in oxyHb-treated BV2 cells with c-Jun knockdown (via siRNA transfection). **(J)** Real-time PCR analysis of METTL3 in oxyHb-treated BV2 cells with c-Jun knockdown (n = 3 biological replicates, one-way ANOVA). **(K)** Protein abundance of c-Jun and METTL3 in oxyHb-treated BV2 cells with c-Jun overexpression (via transfection with Myc-c-Jun plasmid). **(L)** Real-time PCR analysis of METTL3 in oxyHb-treated BV2 cells with c-Jun overexpression (n = 3 biological replicates, one-way ANOVA). Statistical significance is indicated as follows: *: p < 0.05, **: p < 0.01, ***: p < 0.001.

c-Jun (an AP-1 co-factor) is known to regulate METTL3 transcriptional activity (26). On the basis of the correlation between c-Jun and METTL3, we examined the expression of c-Jun in different regions and its correlation with microglial activation status. In a mouse model of ICH, c-Jun was highly expressed in microglia-derived macrophages (**Figures 2A, B**). Furthermore, METTL3 and c-Jun mRNA expression and protein abundance were increased in a time-dependent manner in an oxyHb-induced ICH cell model (**Figures 2B, C**). We also found that the mRNA expression of c-Jun increased in response to OxyHb in a time-dependent manner, which preceded the increase in METTL3 (**Figures 2C, D**). Similar results were also found at the protein level (**Figures 2E–G**). In addition, multiplex immunofluorescence analysis revealed the marked upregulation of c-Jun and METTL3 expression in microglia-derived macrophages (**Figure 2H**). In BV2 cells, *c-Jun* knockdown via transfection with specific siRNAs significantly reduced both *METTL3* mRNA expression and protein abundance (**Figures 2I, J**); conversely, c-Jun overexpression achieved through plasmid transfection induced METTL3 expression (**Figures 2K, L**). These results suggest that c-Jun governs METTL3 transcriptional activity in microglia-derived macrophages, establishing it as an upstream regulator that drives METTL3 upregulation during oxyHb-induced neuroinflammation post-ICH.

### METTL3 promotes the inflammatory response in oxyHb-treated BV2 cells

**Figure 3:**
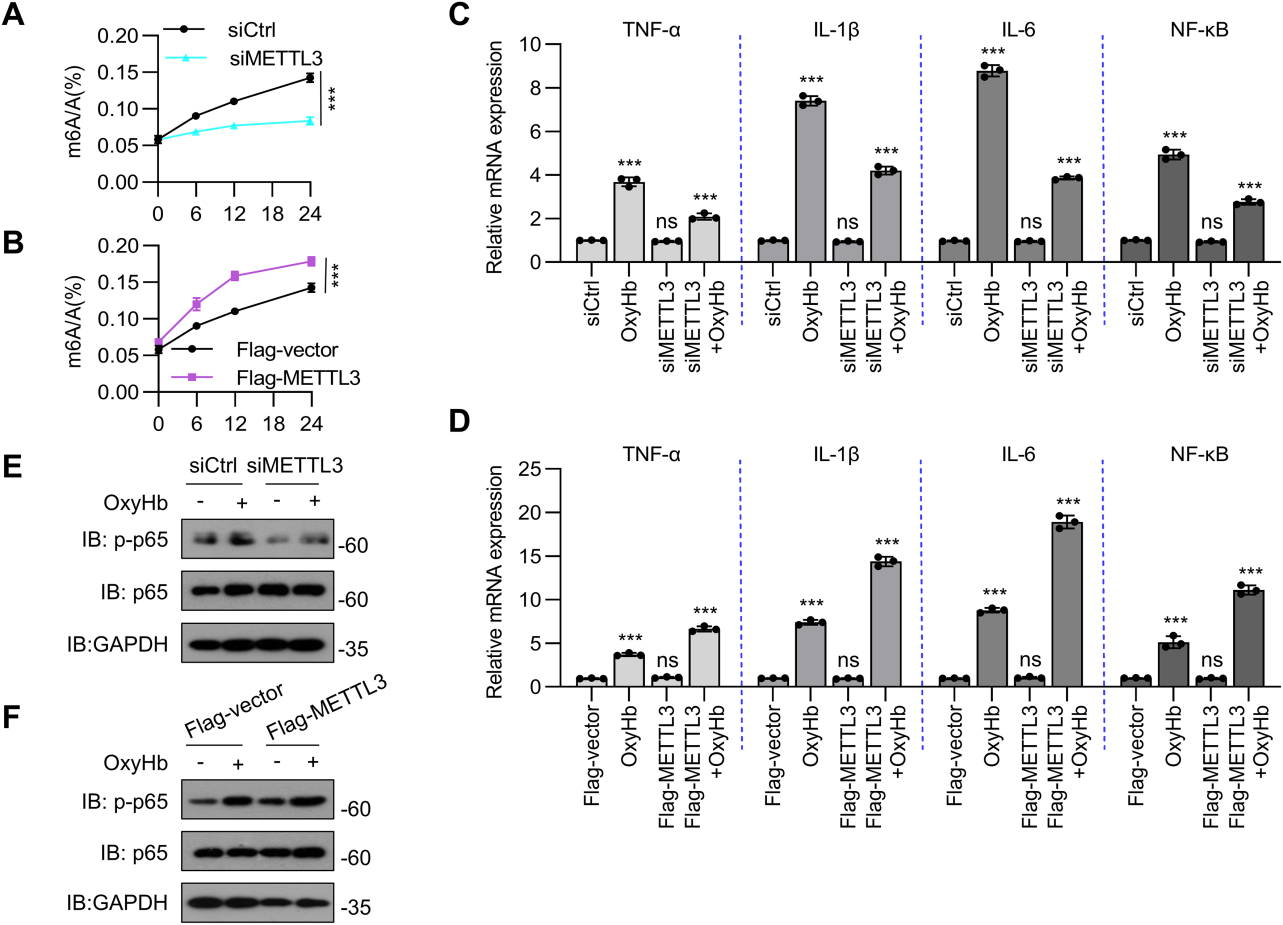
METTL3 enhances inflammatory response in BV2 cells treated with oxyHb stimuli. **(A)** An m6A ELISA assessed m6A mRNA methylation in oxyHb-treated knockdown METTL3 BV2 cells (n=3 biological replicates, *t*-test). **(B)** An m6A ELISA assessed m6A mRNA methylation in oxyHb-treated overexpression METTL3 BV2 cells (via transfection with Flag-METTL3 plasmid)(n = 3 biological replicates, t-test). **(C)** Real-time PCR analysis of expression of inflammatory cytokines and chemokines (TNF-, IL-1β, IL-6, and NF-κB) in oxyHb-treated BV2 cells with and without METTL3 knockdown (n = 3 biological replicates, one-way ANOVA). **(D)** Real-time PCR analysis of inflammatory cytokines (TNF-, IL-1β, IL-6, and NF-κB) in oxyHb-treated BV2 cells with and without METTL3 overexpression (n = 3 biological replicates, one-way ANOVA). **(E, F)** Protein abundance of p-p65 and p65 in oxyHb-treated BV2 cells with METTL3 knockdown or overexpression. Statistical significance is indicated as follows: ***: p < 0.001.

Next, we evaluated the detailed function of METTL3 in BV2 cells in response to inflammatory stimuli. METTL3 was knocked down or overexpressed in BV2 cells via transfection with siRNA or an overexpression plasmid, respectively. The oxyHb–induced increases in m6A modifications were attenuated by *METTL3* knockdown and further increased by METTL3 overexpression (**Figures 3A, B**). TNF-α, IL-1β, and IL-6 are master proinflammatory cytokines that orchestrate innate immune responses, and NF-κB serves as the principal transcriptional regulator governing their expression. Upon inflammatory challenge, TNF-α initiates early-phase inflammation through endothelial activation and leukocyte recruitment (27); IL-1β amplifies immune cascades via pyroptosis induction and acute-phase protein production (28); and IL-6 mediates late-phase responses, including fever and B-cell differentiation (29). Critically, these molecules operate within a self-reinforcing signaling circuit: NF-κB directly transactivates the Tnf, Il1b, and Il6 genes (5); TNF-α and IL-1β reciprocally activate NF-κB through the TNFR/IL-1R-TRAF6-IKK pathways (30); and IL-6 enhances NF-κB sensitivity via JAK-STAT3 crosstalk (31). This axis drives pathological inflammation in neurological disorders, making these molecules critical diagnostic markers. Quantitative real-time PCR analysis demonstrated that siRNA-mediated *METTL3* knockdown in BV2 microglia significantly attenuated oxyhemoglobin-induced proinflammatory responses. Specifically, *METTL3* depletion reduced the mRNA expression of the key cytokines TNF-α, IL-1β and IL-6 (**Figure 3C**). Concurrently, *METTL3* knockdown also reduced the mRNA expression of the transcription factor NF-κB after oxyHb induction (**Figure 3C**). In contrast, the overexpression of METTL3 promoted the oxyHb-induced inflammatory response (**Figure 3D**). In the canonical NF-κB pathway, the p65 (RelA) subunit is primarily phosphorylated at Ser276 and Ser536. Phosphorylation at Ser536 (mediated by IKKβ) is critical for enhancing the DNA-binding affinity of p65 and recruiting coactivators, thereby potentiating the transcriptional activation of proinflammatory genes (e.g., TNF-α and IL-6). Phosphorylation at Ser276 promotes p65 nuclear retention and synergizes with Ser536 phosphorylation to amplify NF-κB-dependent gene expression (32). Therefore, to validate the proinflammatory role of METTL3 in oxyHb-treated BV2 cells, we measured the phosphorylation level of p65 in oxyHb-stimulated BV2 cells. Compared with oxyHb-stimulated BV2 cells, METTL3 knockdown in oxyHb-stimulated BV2 cells suppressed p65 nuclear factor κB (p65 NF-κB) phosphorylation (**Figure 3E**); conversely, the overexpression of METTL3 promoted the phosphorylation of p65 NF-κB (**Figure 3F**). These results suggest that METTL3 might promote oxyHb-induced neuroinflammation in BV2 microglia by enhancing m^6^A modifications, thereby activating the NF-κB signaling pathway (as reflected by p65 phosphorylation) and increasing the levels of proinflammatory cytokines (TNF-α, IL-1β, and IL-6).

### ETV4 is a direct target of METTL3-mediated m6A modification

**Figure 4:**
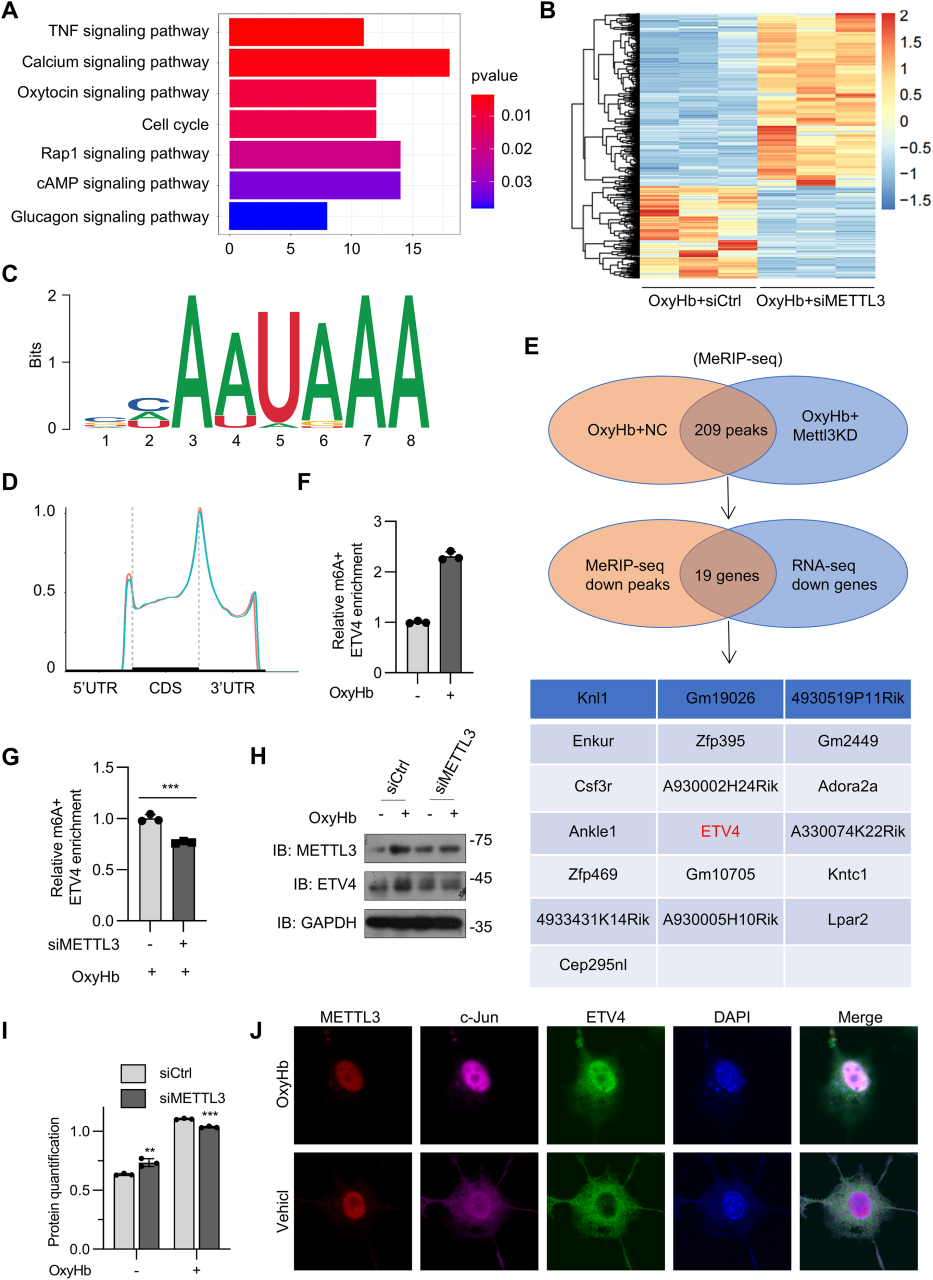
ETV4 is a direct target of METTL3-mediated m6A modification. **(A)** KEGG enrichment analysis cor-related with m6A modification in mice with collagenase-induced ICH compared to control. **(B)** Heatmap of RNA-seq analysis showing differentially expressed genes in oxyHb-stimulated BV2 cells with or without METTL3 knockdown. **(C)** Predominant consensus motif “RRACH” was detected in oxyHb-stimulated BV2 cells with and without METTL3 in m6A seq. **(D)** Density distribution of m6A peaks across mRNA transcripts. **(E)** MeRIP sequencing identified diminished m6A peaks in oxyHb-stimulated BV2 cells with or without METTL3 knockdown. Filtering the diminished m6A peaks with differentially expressed genes in oxyHb-stimulated BV2 cells with or without METTL3 knockdown identified ETV4 as a direct target of METTL3. **(F, G)** MeRIP-qPCR analysis of alterations in the m6A modifications of ETV4 genes in oxyHb-stimulated BV2 cells with or without METTL3 knockdown (n = 3 biological replicates, t test). **(H, I)** Western blot analysis of ETV4 and METTL3 after METTL3 knockdown in oxyHb-treated BV2 **(H)**, the the band density of ETV4 was quantified by **(J)** (n = 3 biological replicates, one-way ANOVA) **(I)**. **(J)** Nuclear co-localization of ETV4, c-Jun, and METTL3 in oxyHb-treated BV2 microglial cells. Statistical significance is indicated as follows: **: p < 0.01, ***: p < 0.001.

We performed methylated RNA immunoprecipitation sequencing (MeRIP-seq) analysis of ICH mouse brains three days after collagenase IV injection. Kyoto Encyclopedia of Genes and Genomes (KEGG) enrichment analysis revealed that m6A methylation was closely related to inflammatory pathways (**Figure 4A**). To identify specific targets of METTL3 in ICH, we conducted MeRIP-seq and RNA sequencing (RNA-seq) analyses using oxyHb-stimulated BV2 cells with or without METTL3 silencing (**Figure 4B**). According to the MeRIP-seq analysis, m6A modifications were typically located in a consensus “RRACH” motif (R = G or A, H = A, C, or U) (**Figure 4C**), and m6A peaks were particularly abundant in the vicinity of 3′ untranslated regions (3′ UTRs) near stop codons (**Figure 4D**), as previously reported. MeRIP-seq analysis revealed that the expression of 209 peaks decreased in oxyHb-stimulated BV2 cells with METTL3 knockdown (**Figure 4E**). Next, we investigated whether the altered m6A peaks were associated with the differentially expressed genes. Filtering the 209 diminished m6A peaks with the downregulated genes resulted in the identification of 19 potential genes (**Figure 4E**). From these candidates, we prioritized ETV4 for further investigation because, as a transcription factor of the ETS family, it has been implicated in driving pro-inflammatory responses in other disease contexts (34). This established role made it a plausible and high-value candidate for mediating microglial activation in ICH. Hence, we focused on ETV4 as a target of METTL3. As a member of the PEA3 subfamily within the ETS transcription factor family, ETS translocation variant 4 (ETV4) regulates pleiotropic biological processes, including cellular differentiation, proliferation, development, and apoptosis (33). Critically, ETV4 functions as a transcriptional activator that directly promotes the expression of TNF-α and MAPK11, thereby driving macrophage infiltration and exacerbating hepatic inflammation (34). Nevertheless, the functional role of ETV4 in the pathological progression of intracerebral hemorrhage (ICH) remains elusive. According to the MeRIP-seq data, one m6A peak was detected around the stop codon of ETV4 mRNA in oxyHb-stimulated BV2 cells, which was diminished upon METTL3 knockdown (**Figure 4E**). Hence, we focused on ETV4 as a target of METTL3. By applying MeRIP–qPCR (quantitative PCR), we found that ETV4 mRNA was enriched by the m6A antibody (**Figure 4F**). m6A methylation of *ETV4* mRNA was induced in oxyHb-treated BV2 cells and was inhibited when *METTL*3 was knocked down (**Figure 4G**). Western blot analysis further confirmed that ETV4 abundance was increased in oxyHb-treated BV2 cells and suppressed following *METTL3* knockdown (**Figures 4H, I**). In oxyHb-treated BV2 cells, ETV4, c-Jun, and METTL3 colocalized in the nucleus (**Figure 4J**). Collectively, these results indicate that ETV4 might be a potential target of METTL3-mediated m6A modification.

### IGF2BP2 enhances ETV4 mRNA stability in an m6A-dependent manner

**Figure 5:**
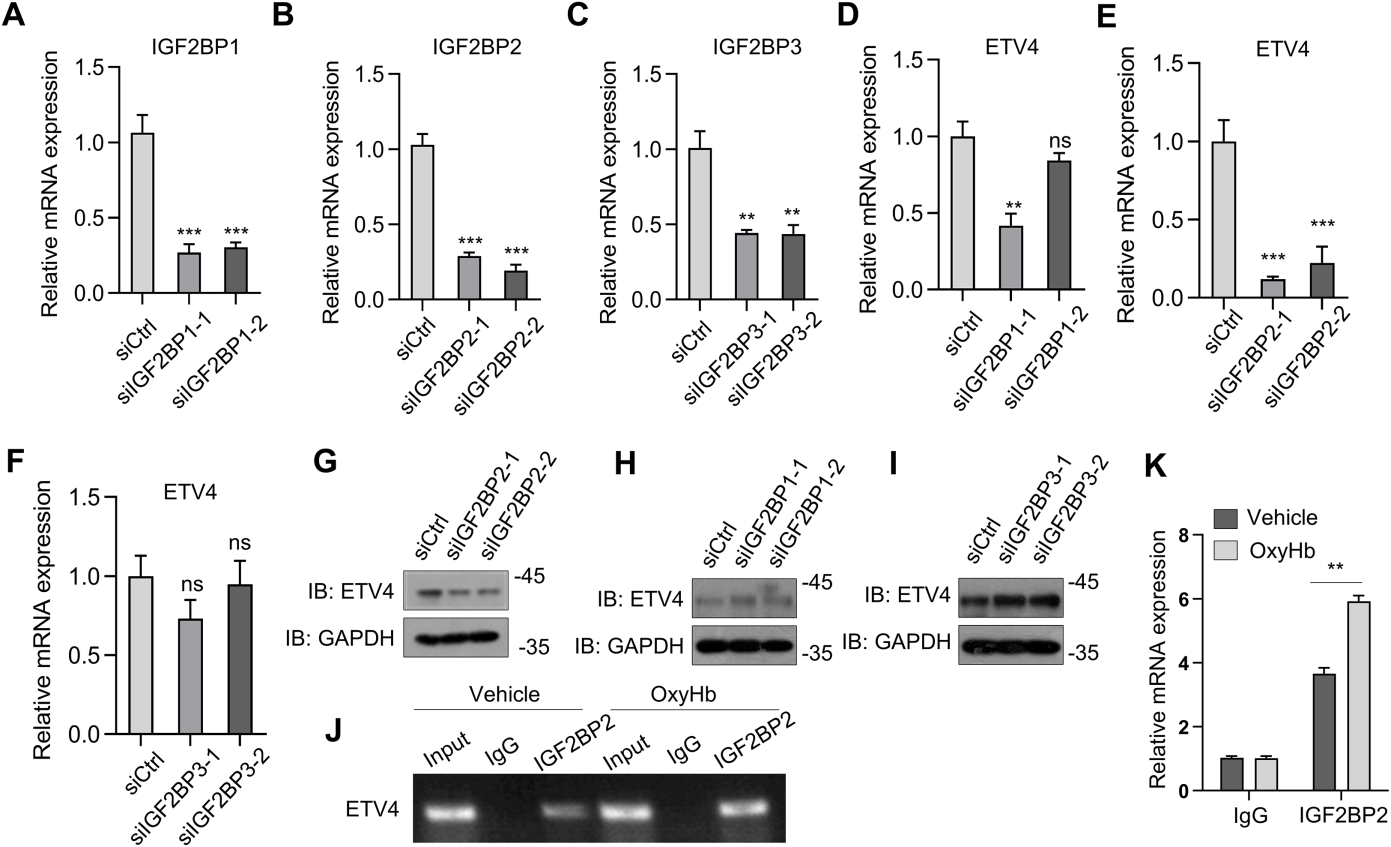
ETV4 serves as a target of METTL3 via an IGF2BP2-dependent mechanism. **(A–C)** Real-time PCR analysis of IGF2BP1 **(A)**, IGF2BP2 **(B)** and IGF2BP3 **(C)** in BV2 cells (n = 3 biological replicates, one-way ANOVA). **(D–F)** Real-time PCR analysis of ETV4 in BV2 cells with IGF2BP1 **(D)**, IGF2BP2 **(E)** and IGF2BP3 **(F)** knockdown (via siRNA transfection) (n = 3 biological replicates, one-way ANOVA). **(G–I)** Western blot analysis of ETV4 after IGF2BP1 **(G)**, IGF2BP2 **(H)** and IGF2BP3 **(I)** inhibition in BV2 cells (n = 3 biological replicates, one-way ANOVA). **(J, K)** Agarose electrophoresis **(J)** and real-time PCR analysis **(K)** of RIP assays in oxyHb-treated BV2 cells showing the direct binding between the IGF2BP2 protein and ETV4 mRNA (n = 3 biological replicates, one-way ANOVA). Statistical significance is indicated as follows: **: p < 0.01, ***: p < 0.001.

Given that m^6^A methylation enhances ETV4 protein stability (**Figure 4H**), we posit that METTL3-mediated m^6^A modification stabilizes ETV4 mRNA transcripts. Although m^6^A marks are typically recognized by decay-promoting readers (e.g., YTHDF2), the insulin-like growth factor 2 binding protein (IGF2BP) family counteracts this process by competitively binding m^6^A sites and shielding transcripts from degradation machinery—specifically by blocking recruitment of the CCR4-NOT deadenylase complex—thereby extending the target mRNA half-life (35). Next, considering that IGF2BP family members play key roles in mediating the stability and translation of m6A-modified mRNAs, we assessed the involvement of IGF2BP1/2/3 in *ETV4* mRNA stabilization. Two specific siRNAs were designed against each target of *IGF2BP1*, *IGF2BP2*, and *IGF2BP3*, and the knockdown efficiency of these constructs was confirmed (**Figures 5A–C**). We found that *IGF2BP2* depletion markedly suppressed ETV4 mRNA expression but that the knockdown of *IGF2BP1* or *IGF2BP3* had a limited effect (**Figures 5D–F**). Those results were confirmed by western blot analysis (**Figures 5G–I**). RNA immunoprecipitation (RIP) analysis with an anti-IGF2BP2 antibody confirmed the interaction between IGF2BP2 and *ETV4* mRNA in oxyHb-treated BV2 cells (**Figures 5J, K**). These results suggest that in oxyHb-stimulated BV2 microglia, IGF2BP2 acts as an m^6^A reader protein that recognizes m^6^A-modified ETV4 transcripts, thereby increasing their mRNA stability.

### The METTL3–ETV4 axis modulates microglial apoptosis in intracerebral hemorrhage pathogenesis

**Figure 6:**
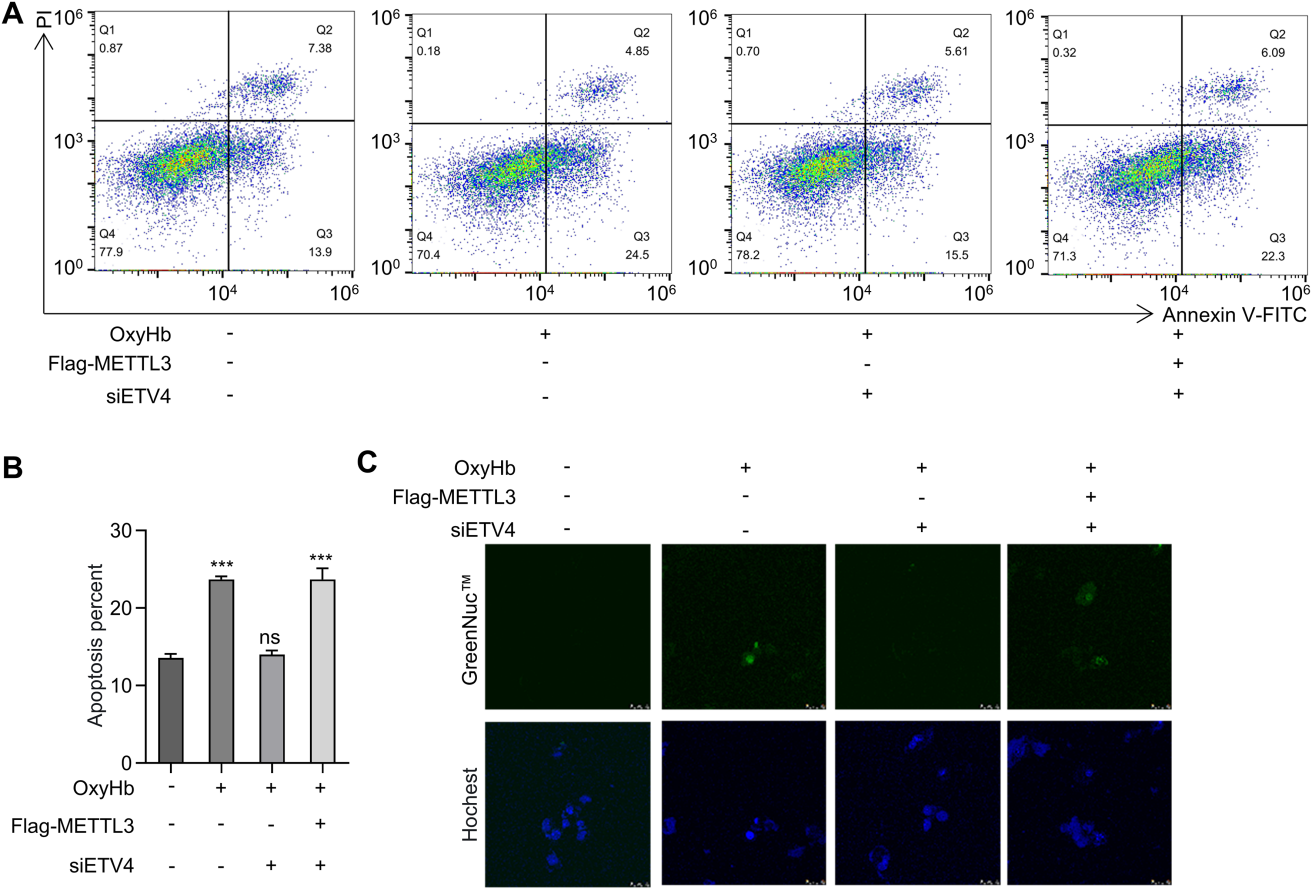
ETV4 knockdown protects against apoptosis in oxyHb-treated BV2 cells. **(A)** Flow cytometry analysis of apoptosis in microglial cells under different treatment conditions. The treatments include: absence (–) or presence (+) of OxyHb, absence (–) or presence (+) of Flag‐METTL3, and absence (–) or presence (+) of siETV4. Annexin V‐FITC staining was used to detect apoptotic cells. Q2 quadrant represents late apoptotic/necrotic cells, and Q4 represents early apoptotic cells. **(B)** Quantitative analysis of the early apoptosis percentage (Annexin V^+^/PI^-^cells) from flow cytometry data (n=3 biological replicates, one-way ANOVA). **(C)** Fluorescence microscopy images showing GreenNuc™ (upper panels, detecting Cleaved-Caspase3) and Hoechst staining (lower panels, counterstaining nuclei) in microglial cells.

To elucidate the functional significance of ETV4 in microglial apoptosis, we performed targeted *ETV4* mRNA knockdown in oxyHb-treated BV2 microglia using siRNA. The results from the quantitative analysis of apoptosis using Annexin V-FITC/PI dual staining coupled with flow cytometry revealed that compared with the control condition, oxyHb stimulation significantly increased the proportion of early apoptotic cells (Annexin V^+^/PI^-^) to 24.5%. Conversely, *ETV4* knockdown via siRNA-ETV4 + oxyHb reduced the early apoptosis rate to 15.5%, which was significantly lower than that in the oxyHb group (P < 0.001). These findings indicate that the downregulation of ETV4 expression effectively suppresses oxyHb-induced microglial apoptosis. To further investigate the role of METTL3 within this regulatory axis, cotransfection with a Flag-METTL3 plasmid and *ETV4* siRNA restored the early apoptosis rate to 22.3%. This rate was significantly greater than that observed with *ETV4* knockdown alone (P < 0.001), demonstrating that METTL3 overexpression specifically reversed the antiapoptotic protective effect conferred by ETV4 deficiency (**Figures 6A, B**). Given that cleaved caspase-3, the activated form of the key executioner caspase, serves as a direct indicator of apoptosis progression (36), we assessed its levels in live cells across treatment groups. Consistent with the above findings, *ETV4* knockdown combined with METTL3 overexpression resulted in the marked accumulation of cleaved caspase-3 relative to that in the control (**Figure 6C**). Collectively, these results establish the METTL3–ETV4 axis as a critical pathway regulating microglial apoptosis in the pathophysiology of ICH.

## Discussion

This study reveals for the first time that in the microenvironment of ICH, METTL3 expression is significantly upregulated in microglia. Through catalyzing m6A modifications on ETV4 mRNA (mainly enriched in the 3’UTR with RRACH motifs), METTL3 recruits the m6A reader IGF2BP2, thereby increasing the stability and protein expression levels of ETV4 mRNA. this regulatory mechanism is associated with a pro-inflammatory microglial phenotype, including a significant increase in the secretion of factors such as TNF-α and IL-6 and the upregulation of M1 polarization-related gene expression, ultimately disrupting the balance between anti-inflammatory and proinflammatory responses. This axis promotes neuroinflammation, primarily through NF-κB activation, and concurrently induces microglial apoptosis, as evidenced by caspase-3 activation. Cell experiments revealed that the inhibition of METTL3 or knockdown of ETV4 can significantly alleviate neuroinflammation and brain tissue damage caused by intracerebral hemorrhage, suggesting a causal relationship between this regulatory axis and disease progression. Although our gain- and loss-of-function studies demonstrate that ETV4 upregulation is both necessary and sufficient for driving pro-inflammatory responses, the precise molecular mechanism remains to be fully elucidated. As an ETS-family transcription factor, ETV4 could potentially regulate inflammatory gene expression directly by binding to promoter regions of genes like Tnf or Il6, or indirectly through intermediate regulators. Future studies employing Chromatin Immunoprecipitation (ChIP) assays will be essential to definitively identify the direct transcriptional targets of ETV4 in microglia and establish a direct mechanistic link. The methodological advantage of this study lies in the combination of molecular biology and cell function experiments. MeRIP-qPCR analysis verified the m6A modification level of ETV4. RNA immunoprecipitation (RIP) experiments confirmed the direct binding between IGF2BP2 and ETV4. The use of primary microglial cell culture models ensured the cellular specificity of the mechanism and avoided interference from complex microenvironments *in vivo*.

Despite these significant insights, our study has limitations that should be considered. The primary reliance on the BV2 microglial cell line, while instrumental for mechanistic dissection, cannot replicate the intricate multicellular crosstalk (e.g., with astrocytes, neurons, and infiltrating immune cells) that characterizes the ICH milieu *in vivo*. Therefore, caution is warranted when extrapolating these findings to the more complex pathophysiology *in vivo*. The dynamic spatiotemporal regulation of the METTL3-ETV4-IGF2BP2 axis within this complex environment remains to be fully elucidated. For example, the heterogeneity of microglial subpopulations and their differential responses to hemorrhagic injury could introduce variability in METTL3 expression and m6A modification patterns. Additionally, the downstream effector mechanisms of ETV4, particularly the interplay of ETV4 with other transcription factors and epigenetic regulators involved in microglial polarization and apoptosis, warrant further investigation. The potential crosstalk between ETV4 and other signaling pathways, such as the STAT or MAPK cascades, could provide a more comprehensive understanding of the role of ETV4 in neuroinflammation. Furthermore, the contribution of other m6A regulatory proteins, including METTL14, FTO, or ALKBH5, to this process remains unexplored, raising questions about the broader context of m6A epitranscriptomic regulation in ICH pathogenesis.

Here, the therapeutic implications of targeting the METTL3–ETV4 axis are promising, as evidenced by the amelioration of neuroinflammation and tissue damage upon METTL3 inhibition or *ETV4* knockdown. However, the development of clinically viable interventions, such as small-molecule METTL3 inhibitors or IGF2BP2 antagonists, will require rigorous validation in preclinical ICH models. Future research should focus on delineating cell type-specific responses and the temporal dynamics of m6A modifications in the injured brain. Additionally, the potential off-target effects and pharmacokinetic challenges associated with modulating the m6A machinery must be carefully evaluated. Overall, this study reveals a new mechanism by which METTL3 in microglia enhances the stability of ETV4 through IGF2BP2-dependent m6A modification, driving abnormal activation, inflammation imbalance, M2 polarization impairment, and microglia apoptotic hyperactivity, ultimately exacerbating the pathological process of ICH. As a transcription factor, ETV4 likely executes these diverse functions by regulating distinct sets of downstream target genes. For instance, its known role in activating TNF-α and MAPK11 [Ref 34] could directly explain the pro-inflammatory effects, while its potential regulation of pro-apoptotic genes may underlie the caspase-3-dependent apoptosis observed in our study. Future work employing ChIP-seq and transcriptomic analyses in microglia will be crucial to comprehensively identify the direct transcriptional targets of ETV4 responsible for these specific phenotypes in ICH. This study not only advances our understanding of the epitranscriptomic regulation of microglial activation but also opens new avenues for therapeutic strategies aimed at mitigating neuroinflammation and improving outcomes after ICH. Further exploration of this regulatory axis *in vivo* and its integration with other pathological mechanisms will be essential for translating these findings into clinical applications.

## Materials and methods

### Reagents andantibodies

Antibodies included anti-METTL3 (ABclonal, A19079), anti-METTL14 (ABclonal, A8530), anti-WTAP (ABclonal, A14695), anti-FTO (ABclonal, A3861), anti-CD45 (Servicebio, GB113886), antiCD11b (Servicebio, GB15058), anti-c-Jun (Proteintech, 11515-1-AP), anti-Caspase3 (Servicebio, GB11767C), anti-Cleaved-Caspase-3 (Servicebio, GB115733), anti-GAPDH (Servicebio, GB15004), anti-p65 (Servicebio, GB11997), anti-p-p65 (Servicebio, GB113882), anti-ETV4 (proteintech, 10684-1-AP), anti-IGF2BP2 (proteintech, 11601-1-AP). collagenase IV were purchased from Sigma-Aldrich (9001-12-1). OxyHb were purchased from yingxinbio (TX00900).

### Cell cultures and treatments

The murine BV2 microglial cells were obtained from FuHeng Cell Center (Shanghai, China), with identity confirmed by STR profiling and absence of mycoplasma contamination. Cells were maintained in DMEM supplemented with 10% FBS at 37 °C in a 5% CO_2_ atmosphere.

### RNA isolation and quantitative real-time PCR

Total RNA was extracted with TRIzol reagent (Invitrogen), and quantitative PCR was performed using SYBR Green master mix (Toyobo, QPK201). Gene-specific primers (listed in **Supplementary Table S1**) targeted METTL3, METTL14, WTAP, FTO, ALKBH5, c-Jun, TNF-α, IL-1β, IL-6, NF-κB, IGF2BP1-3, ETV4, and β-actin.

### Dot blot assay

Global m^6^A levels were assessed by an RNA dot blot assay. Briefly, total RNA extracted with TRIzol (Invitrogen, 15596018) was spotted onto a nylon membrane (Sigma-Aldrich, GERPN1210B), UV-crosslinked, blocked with 5% nonfat milk, and sequentially incubated with an anti-m^6^A antibody (Abcam) and a secondary antibody. Signal detection was performed using a LI-COR/Odyssey imaging system, with total RNA quantified by methylene blue staining.

### Animal studies

All animal procedures were approved by the Animal Experimentation Ethics Committee of Shandong Provincial Hospital Affiliated to Shandong First Medical University (Approval No. 2024-149) and conducted in accordance with the Guide for the Care and Use of Laboratory Animals. Male C57BL/6J mice (6–8 weeks old, 20–22 g) from the hospital’s Experimental Animal Center were used. The intracerebral hemorrhage (ICH) model was induced by stereotactic intrastriatal injection of collagenase IV (Sigma-Aldrich, 9001-12-1) at 10 mg/kg. Sham-operated mice received an equal volume of saline. All mice were sacrificed under anesthesia 3 days post-injection for brain tissue collection and analysis (paraffin embedding, molecular assays). Each group contained at least three mice.

### Methylated RNA immunoprecipitation sequencing

MeRIP-seq was performed as described (5). Briefly, total RNA was extracted with TRIzol reagent (Invitrogen), and poly(A) mRNA was enriched using the Dynabeads™ mRNA Purification Kit (Invitrogen). The mRNA was fragmented to ~100 nt using RNA Fragmentation Reagents (Invitrogen, AM8740). A portion (10%) of the fragmented RNA was saved as input. The remainder was incubated overnight at 4 °C with an anti-m6A antibody (Synaptic Systems, 202003) preconjugated to Dynabeads Protein A (Life Technologies) in IP buffer. The bead-antibody-RNA complexes were sequentially washed with high-, middle-, and low-salt buffers. Immunoprecipitated RNA was eluted with TRIzol, and both input and IP samples were submitted to RiboBio (China) for library preparation and sequencing.

### MeRIP-qPCR

MeRIP-qPCR was performed as previously described (5). Briefly, poly(A)+ RNA was purified from 50 μg of total RNA using the Dynabeads™ mRNA Purification Kit (Invitrogen, 61006). Pierce Protein A/G Magnetic Beads (Thermo Fisher, 88803) were pre-incubated with 5 μg of anti-m6A antibody (Synaptic Systems, 202003) to form conjugates. The antibody-bead complexes were then incubated with the purified RNA in IP buffer. After washing, the m6A-enriched RNA was precipitated and analyzed by qPCR. Enrichment was calculated relative to the input control.

### Brain histology and immunohistochemistry

Immunohistochemical staining was performed on 4-μm paraffin-embedded mouse brain sections following standard protocols. Sections were incubated overnight at 4 °C with primary antibodies against METTL3 (ABclonal, A19079) and c-Jun (Proteintech, 11515-1-AP), followed by a 2-hour incubation with secondary antibodies at room temperature. Staining was visualized using diaminobenzidine (DAB) with hematoxylin counterstaining, and images were captured with a Leica Aperio GT450 microscope.

### Immunofluorescent staining

For immunofluorescence, deparaffinized brain sections from ICH model mice were incubated overnight at 4 °C with antibodies against METTL3 (ABclonal, A19079), c-Jun, CD45, and CD11b (Servicebio, GB11515, GB113886, GB15058), followed by a 2-hour incubation with secondary antibodies at room temperature. Images were acquired using a Leica Aperio GT450 microscope. For the cell model, BV2 cells were fixed and immunostained as previously described (37), and analyzed with a confocal laser-scanning microscope (Leica TCS-SPE).

### RNA immunoprecipitation

RNA immunoprecipitation (RIP) was performed using the PureBinding RIP Kit (Geneseed) per the manufacturer’s instructions. Briefly, BV2 cell lysates were incubated with magnetic beads coated with 5 μg of anti-IGF2BP2 (Proteintech, 11601-1-AP) or control mouse IgG (Millipore) antibody for 4 hours at room temperature. After six washes, the co-precipitated RNA was isolated via proteinase K digestion. The enrichment of ETV4 mRNA was quantified by qPCR.

### Quantification of m6A modifications

Total RNA was extracted using TRIzol reagent (Invitrogen, 15596018) and treated with DNase I (Sigma-Aldrich, 04716728001). RNA quality was confirmed by NanoDrop spectrophotometry. Global m6A methylation levels were colorimetrically quantified using the EpiQuik m6A RNA Methylation Quantification Kit (Epigentek) according to the manufacturer’s protocol, by measuring the absorbance at 450 nm with 200 ng of input RNA.

### Knockdown by short interfering RNA

Plasmid or siRNA transfection was carried out using Lipofectamine 3000 (Invitrogen, L3000001) according to the manufacturer’s protocol, and analyses were performed 48–72 hours post-transfection. The siRNA sequence targeting ETV4 was 5′-CUCGCUGCGAUACUAUUAUTT-3′(sense) and 5′-AUAAUAGUAUCGCAGCGAGTT-3′(antisense). Sequences for METTL3 and c-Jun siRNA were as previously published (5).

### Apoptosis assay

Cell apoptosis was assessed 72 hours post-transfection using an Annexin V-FITC/PI Apoptosis Detection Kit (Beyotime, C1062) according to the manufacturer’s instructions. Briefly, harvested cells were resuspended in binding buffer containing Annexin V-FITC and PI, and then analyzed by flow cytometry (FACScan; BD Biosciences). Data were processed with BD FACSDiva software (v6.1.3) to quantify the percentages of viable, necrotic, and apoptotic cells. Experiments were performed in triplicate. For data analysis, the percentage of early apoptotic cells was defined as the Annexin V^+^/PI^-^population (Q4).

### Statistical analysis

Statistical analyses were performed using SPSS 23.0. Data are presented as mean ± SEM after confirming normal distribution with the Shapiro–Wilk test. Differences between groups were assessed by an unpaired t-test or one-way ANOVA followed by Tukey’s *post hoc* test, using GraphPad Prism 9.0 for graph generation. A p-value < 0.05 was considered statistically significant.

## Data Availability

The data presented in the study are deposited in the BioProject repository, accession number PRJNA1394618.
